# Community norms of the Muscle Dysmorphic Disorder Inventory (MDDI) among gender minority populations

**DOI:** 10.1186/s40337-021-00442-4

**Published:** 2021-07-14

**Authors:** Jason M. Nagata, Emilio J. Compte, F. Hunter McGuire, Jason M. Lavender, Tiffany A. Brown, Stuart B. Murray, Annesa Flentje, Matthew R. Capriotti, Micah E. Lubensky, Juno Obedin-Maliver, Mitchell R. Lunn

**Affiliations:** 1 Department of Pediatrics, University of California, 550 16th Street, 4th Floor, Box 0110, San Francisco, CA 94158 USA; 2 Eating Behavior Research Center, School of Psychology, Universidad Adolfo Ibáñez, Santiago, Chile; 3 Research Department, Comenzar de Nuevo Treatment Center, Monterrey, Mexico; 4 The Brown School, Washington University in St. Louis, St. Louis, MO USA; 5 Military Cardiovascular Outcomes Research Program (MiCOR), Department of Medicine, Uniformed Services University of the Health Sciences, Bethesda, MD USA; 6 The Metis Foundation, San Antonio, TX USA; 7 Department of Psychiatry, University of California, San Diego, CA USA; 8 San Diego State University Research Foundation, San Diego, CA USA; 9 Department of Psychiatry and Behavioral Sciences, University of Southern California, Los Angeles, CA USA; 10 Department of Community Health Systems, University of California, San Francisco, San Francisco, CA USA; 11 Alliance Health Project, Department of Psychiatry and Behavioral Sciences, University of California, San Francisco, San Francisco, CA USA; 12 The PRIDE Study/PRIDEnet, Stanford University School of Medicine, Stanford, CA USA; 13 Department of Psychology, San José State University, San Jose, CA USA; 14 Department of Obstetrics and Gynecology, Stanford University School of Medicine, Stanford, CA USA; 15 Department of Epidemiology and Population Health, Stanford University School of Medicine, Stanford, CA USA; 16 Division of Nephrology, Department of Medicine, Stanford University School of Medicine, Stanford, CA USA

**Keywords:** Muscle dysmorphia, Muscle dysmorphic disorder, Body image, Body dissatisfaction, Body dysmorphia, Transgender, Gender non-conforming, Genderqueer, Gender minority, LGBTQ

## Abstract

**Purpose:**

Representing the pathological extreme pursuit of muscularity, muscle dysmorphia (MD) is characterized by a pervasive belief or fear around insufficient muscularity and an elevated drive for muscularity. Despite evidence of heightened body image-related concerns among gender minority populations, little is known about the degree of MD symptoms among gender minorities, particularly based on Muscle Dysmorphic Disorder Inventory (MDDI) scores. The objective of this study was to assess community norms of the MDDI in gender-expansive people, transgender men, and transgender women.

**Method:**

Data from participants in The PRIDE Study, an existing study of health outcomes in sexual and gender minority people, were examined. We calculated means, standard deviations, and percentiles for the MDDI total and subscale scores among gender-expansive people (i.e., those who identify outside of the binary system of man or woman; *n* = 1023), transgender men (*n* = 326), and transgender women (*n* = 177). The Kruskal-Wallis test was used to assess group differences and post hoc Dunn’s tests were used to examine pairwise differences.

**Results:**

Transgender men reported the highest mean MDDI total score (30.5 ± 7.5), followed by gender-expansive people (27.2 ± 6.7), then transgender women (24.6 ± 5.7). The differences in total MDDI score were driven largely by the Drive for Size subscale and, to a lesser extent, the Functional Impairment subscale. There were no significant differences in the Appearance Intolerance subscale among the three groups.

**Conclusions:**

Transgender men reported higher Drive for Size, Functional Impairment, and Total MDDI scores compared to gender-expansive people and transgender women. These norms provide insights into the experience of MD symptoms among gender minorities and can aid researchers and clinicians in the interpretation of MDDI scores among gender minority populations.

## Plain English summary

Transgender people have a gender identity or expression that differs from what is typically associated with the sex assigned to them at birth. Gender-expansive refers to gender identities that do not fit within the binary (woman/man) gender system. We asked gender-expansive people, transgender men, and transgender women in The PRIDE Study to fill out a widely used survey about muscle dysmorphia, a condition in which a person worries they are not muscular enough. The results showed that transgender men had the highest muscle dysmorphia symptoms, followed by gender-expansive people, then transgender women. These findings can help clinicians and researchers in interpreting a survey assessing muscle dysmorphia in gender-expansive people, transgender men, and transgender women. Characterizing muscle dysmorphia symptoms in these populations is an important step to improve mental wellness among transgender and gender-expansive people.

## Introduction

Muscle dysmorphia (MD), a specifier of Body Dysmorphic Disorder (BDD) in the Fifth Edition of the Diagnostic and Statistical Manual of Mental Disorders (DSM-5), is characterized by an excessive preoccupation with muscularity and the belief that one’s body or body parts are insufficiently muscular [1]. MD involves a host of social and functional impairments arising from time-consuming behavioral aspects of MD (e.g., excessive exercise, mirror checking, disordered eating behaviors) and shame about perceived physical appearance flaws [2–4]. In addition, individuals with MD symptoms often have comorbid psychiatric diagnoses and symptoms, including mood and anxiety disorders, substance use disorders, suicidal behaviors, and disordered eating behaviors [2, 3, 5]. These impairments highlight the need to investigate populations that may be at higher risk for the development of MD symptoms, including gender minorities.

Most prior MD research has been conducted using samples of cisgender men (i.e., individuals who identify as a man and were assigned male at birth) [3, 6, 7]. As a result, there is limited empirical knowledge about experiences of MD among people with other genders and gender identities, including transgender and gender-expansive (TGE) populations. Transgender people have a gender identity or expression that differs from those traditionally associated with the sex assigned to them at birth. (See Table 1 for additional clarification on assigned sex at birth and gender identity among transgender men and transgender women, respectively.) Gender-expansive refers to a spectrum of gender identities which exist outside the gender binary (i.e.*,* man, woman), including, but not limited to, individuals with no particular or multiple gender identities and those whose gender identity shifts over time or in different contexts. The current study reports on transgender men, transgender women, and gender-expansive individuals. For the purposes of this research, transgender men and transgender women indicated having a gender that aligned with the gender binary, while gender-expansive individuals indicated having a gender or genders that exist outside of the gender binary.

**Table 1:** Explanation of Classification of Participants

Population	Gender identity	Sex assigned at birth
Cisgender man	man (exclusively)	male
Cisgender woman	woman (exclusively)	female
Transgender man	man, transgender man, or transmasculine (write-in)^a^	female
Transgender woman	woman, transgender woman, or transfeminine (write-in)^b^	male
Gender-expansive person	Included: genderqueer, multiple gender identities, another gender identity, non-binary, nonconforming, genderfluid, intersex, two-spirit, agender, bigender. This category included anyone not classified as cisgender man, cisgender woman, transgender man, or transgender woman.	

^a^Includes any combination of man, transgender man, and/or transmasculine, but not other gender identities

^b^Includes any combination of woman, transgender woman, and/or transfeminine, but not other gender identities

Stemming from individual, interpersonal, and structural stigma, the higher prevalence of adverse health outcomes among TGE populations relative to cisgender populations is well-documented [8]. In particular, the gender minority stress framework posits that TGE people may be more likely to experience poor psychological health due to a variety of social factors, including internalized transphobia (i.e., discomfort with one’s TGE identity arising from an internalization of societal gender norms that privilege cisgender gender identities and expressions), gender-related discrimination, and gender identity non-affirmation [9, 10].

Due to diverse social experiences and community-specific body image norms, gender minority communities are not monolithic, and some groups may be at differential risk for the development of MD symptoms. Emerging research conducted in Italy indicates that transgender men report an equally high drive for muscularity as cisgender men, with nonbinary individuals, transgender women, and cisgender women reporting lower muscularity concerns [11]. Similar to cisgender women, transgender women may experience lower muscularity concerns due to feminine body norms placing greater emphasis on thinness [12]. Moreover, gender-expansive individuals may be disproportionately exposed to certain social stressors and psychological comorbidities that, in turn, may place them at higher risk for body image concerns. For instance, relative to cisgender and binary transgender men and women, gender-expansive individuals report higher lifetime experiences of psychological distress and self-harm behaviors, as well as being targeted for interpersonal violence and discrimination such as sexual assault and harassment [13]. Taken together, these diverse factors highlight the importance of the nature and severity of MD symptoms across different gender minority groups.

Though other MD symptom measures exist (e.g., the Muscle Dysmorphia Inventory [14], the Muscle Appearance Satisfaction Scale [15]), the Muscle Dysmorphic Disorder Inventory (MDDI) [16] was chosen for the current study, as it is one of the most widely used questionnaires assessing MD symptoms [7]. The MDDI has been psychometrically evaluated in samples of mostly cisgender men from diverse geographic locations and in multiple languages [17–23], and has received psychometric support specifically in samples of cisgender gay men and lesbian women [24]. One recent study used the MDDI to examine MD symptoms among TGE participants from Italy [11]; however, this investigation was limited by a small sample of TGE individuals and did not generate norms for the measure. No prior research has examined the nature and severity of MD symptoms and reported MDDI norms among gender-expansive individuals, transgender men, and transgender women from the community in the United States. Understanding MD symptoms in these populations is critical to the development of gender-inclusive clinical and public health interventions for preventing or mitigating muscularity-oriented body image concerns and related behavioral symptoms (e.g., excessive exercise, disordered eating). Therefore, the purpose of the current study is to describe community norms of the MDDI in three gender minority populations from the United States and to compare MDDI scores across these populations. Consistent with findings from prior research on TGE individuals [14], it was expected that MD symptoms would be highest among transgender men.

## Methods

The current study is based on data from a subsample of participants in The Population Research in Identity and Disparities for Equality (PRIDE) Study. The PRIDE Study is a national online longitudinal cohort study that examines the health and wellbeing of sexual and gender minority adults in the United States. From April 2018 to August 2018, 4285 participants from The PRIDE Study completed a single cross-sectional ‘Eating and Body Image’ survey via any web-enabled device. More information about The PRIDE Study’s research design and recruitment procedures can be found elsewhere [25, 26].

### Inclusion Criteria & Study Population

Inclusion criteria for the current study were completing the ‘Eating and Body Image’ survey (at least 50% of the MDDI, age, and body mass index [BMI] questions); living in the United States or its territories (e.g., Puerto Rico); being aged 18 years or older; being able to read and respond to an English-language survey; and being classified as a gender-expansive person, transgender man, or transgender woman.

Of the 4282 participants from The PRIDE Study, 1653 (38.6%) identified as either a gender-expansive person, a transgender man, or a transgender woman. Of these 1653 participants, 127 (7.7%) were excluded because they completed less than half of the relevant survey items (MDDI) and/or critical characteristics (age, BMI). Among gender subgroups, this resulted in 97 (8.7%) gender-expansive individuals, 26 (7.4%) transgender men, and four (2.2%) transgender women being excluded. The final sample included 1526 participants who were classified as gender-expansive people (*N* = 1023; 67.0%), transgender men (*N* = 326; 21.4%), or transgender women (*N* = 177; 11.6%).

### Participant Recruitment & Informed Consent

Participant recruitment occurred through several methods: community engagement through PRIDEnet (a national network of organizations and individuals to engage sexual and gender minority communities), online advertising through blog posts and newsletters, distribution of The PRIDE Study-branded promotional items, conference and event-based outreach, advertisements on social media, and word-of-mouth. Data were collected on a cloud-based, secure, digital platform [26]. Participants could access the survey platform from any smartphone, tablet, or computer. Compensation was not provided for completing the survey. This study was approved by the Stanford University and University of California, San Francisco Institutional Review Boards, as well as The PRIDE Study’s Research Advisory Committee and Participant Advisory Committee. All participants provided written informed consent to participate.

### Measures

#### Sociodemographic questionnaire

Participants self-reported sociodemographic information, including race, ethnicity, age, country of birth, and education level. We calculated BMI based on self-reported weight and height [weight (kg)/height (m)^2^].

#### Gender Identity & sex Assigned at birth

Participants were able to indicate their current gender identity (check all that apply) with options of “Man,” “Genderqueer,” “Transgender man,” “Transgender woman,” “Woman,” or “Another gender identity” (with the option to specify). Participant’s sex assigned at birth was assessed with the question “What sex were you assigned at birth on your original birth certificate?” with options of “Female” or “Male.” Table 1 describes the classification rules that were applied to form the final samples. Participants in the current study were classified as either: (1) transgender man (man/transgender man/transmasculine [write-in] gender identity and female sex assigned at birth); (2) transgender woman (woman/transgender woman/transfeminine [write-in] gender identity and male sex assigned at birth); or (3) gender-expansive people including genderqueer, multiple gender identities, another gender identity, non-binary, nonconforming, genderfluid, intersex, two-spirit, agender, and bigender (anyone not classified as a cisgender man, cisgender woman, transgender man, or transgender woman).

#### Muscle dysmorphic disorder inventory (MDDI)

The MDDI is a 13-item measure designed to assess symptoms of muscle dysmorphia with individual items rated on a Likert-type scale from 1 (*never*) to 5 (*always*) [16]. The measure provides a total score and three subscale scores: Drive for Size (DFS; 5 items, range 5–25), Appearance Intolerance (AI; 4 items, range 4–20), and Functional Impairment (FI; 4 items, range 4–20). Items are summed to generate the total score and subscale scores, with higher values reflecting a greater severity of MD symptoms. The MDDI has demonstrated evidence of reliability and validity in samples of college-aged men [16] and sexual minority men and women [24, 27]. For participants in this study, item five *(“I think my chest is too small”*) was modified to specify “chest (muscle)”, so as to not confuse “chest” with breast size [24]. The MDDI total and subscale scores demonstrated acceptable to good internal consistency for gender-expansive participants and transgender men, respectively: total score (α = 0.71 and 0.74), DFS (α = 0.81 and 0.82), AI (α = 0.78 and 0.77), and FI (α = 0.83 and 0.83); for transgender women, internal consistency for the MDDI total and subscale scores ranged from questionable to acceptable: total score (α = 0.67), DFS (α = 0.65), AI (α = 0.74), and FI (α = 0.79).

### Data analysis

Results are presented in terms of percentiles, mean (standard deviation), median (interquartile range [IQR]), and percentage. The assumption of normality was not fulfilled among continuous variables (Shapiro-Wilk: *p* < .001), so the Kruskal-Wallis test was used to assess gender group differences (gender-expansive people, transgender men, transgender women) and post hoc Dunn’s tests were used to examine pairwise differences [28]. The chi-square test was used to compare proportions of participants in each group that had an MDDI total score reflecting clinical significance (based on a cutoff score of ≥39 [23]).

The R statistical environment (RStudio, version 3.6.2; R Development Core Team, 2019) was used to conduct analyses. The *psyche* package [29] was used to conduct bivariate analyses. For participants with missing data but greater than 50% of responses to items, missing values were minimal (gender-expansive people: 0.08%; transgender men: 0.07%; transgender women: 0.02%). The *MissMech* package’s nonparametric test of homoscedasticity was used to assess the mechanism of missing data [30]. All missing data were consistent with missing completely at random (*p* > .05); the *mice* package was used to perform data imputation with chained equations multivariate imputation [31]. A two-tailed threshold of *p* < .05 was used for evaluating significance of the pairwise comparisons.

## Results

Table 2 presents participant sociodemographics. Across all three groups, participants were predominantly White and college-educated with mean BMIs ranging from 28.0 to 29.7 kg/m^2^. The mean age for gender-expansive participants and transgender men was similar (30.0 and 30.9 years, respectively), whereas transgender women had a higher mean age of 41.2 years. Norms for the MDDI across the three groups are presented in Table 3.

**Table 2:** Sociodemographic characteristics of gender-expansive people, transgender men, and transgender women from The PRIDE Study

	Gender-Expansive People	Transgender Men	Transgender Women
N	1023	326	177
**Sociodemographic characteristics**	Mean ± SD / %	Mean ± SD / %	Mean ± SD / %
Age, years	30.0 ± 9.9	30.9 ± 9.8	41.2 ± 15.0
Race			
White	80.7%	88.9%	89.5%
Asian/Pacific Islander	2.9%	0.3%	0.6%
Black/African American	1.1%	2.9%	0.0%
Native American	0.3%	0.3%	0.0%
Two or more races	2.7%	0.8%	3.5%
Another Race	12.3%	6.7%	6.4%
Ethnicity: Hispanic/Latino/a	5.4%	3.9%	3.9%
Born in the US	87.6%	89.2%	90.6%
Educational attainment			
College degree or higher	58.1%	56.8%	55.8%
Body mass index (BMI), kg/m^2^	29.7 ± 8.5	28.8 ± 7.4	28.0 ± 6.4

**Table 3:** Distribution of means, standard deviations, medians, interquartile ranges, and percentile ranks for the Muscle Dysmorphic Disorder Inventory (MDDI) among gender-expansive people, transgender men, and transgender women from The PRIDE Study

	Gender-Expansive People (*N* = 1023)				Transgender Men (*N* = 326)				Transgender Women (*N* = 177)			
	MDDI DFS	MDDI AI	MDDI FI	MDDI Total	MDDI DFS	MDDI AI	MDDI FI	MDDI Total	MDDI DFS	MDDI AI	MDDI FI	MDDI Total
*M* (SD)	8.3 (3.9)	12.8 (4.0)	6.1 (3.1)	27.2 (6.7)	10.7 (4.5)	13.2 (4.0)	6.6 (3.3)	30.5 (7.5)	6.1 (2.2)	13.0 (4.0)	5.5 (2.6)	24.6 (5.7)
Range	5–24	4–20	4–20	13–56	5–25	4–20	4–20	13–57	5–22	4–20	4–16	14–43
Percentile rank												
5	5.0	6.0	4.0	17.0	5.0	6.0	4.0	20.0	5.0	6.0	4.0	16.0
10	5.0	7.0	4.0	19.0	5.0	7.5	4.0	22.0	5.0	7.0	4.0	18.0
15	5.0	8.0	4.0	20.0	6.0	9.0	4.0	23.0	5.0	9.0	4.0	19.0
20	5.0	9.0	4.0	21.0	7.0	10.0	4.0	25.0	5.0	10.0	4.0	20.0
25	5.0	10.0	4.0	22.0	7.0	10.3	4.0	25.0	5.0	10.0	4.0	21.0
30	5.0	11.0	4.0	23.0	8.0	11.0	4.0	26.0	5.0	11.0	4.0	21.0
35	6.0	11.0	4.0	24.0	8.0	12.0	4.0	27.0	5.0	11.0	4.0	22.0
40	6.0	12.0	4.0	25.0	9.0	12.0	4.0	28.0	5.0	11.4	4.0	22.4
45	7.0	12.0	4.0	26.0	9.0	13.0	4.3	29.0	5.0	12.0	4.0	23.0
50	7.0	13.0	4.0	27.0	10.0	13.0	5.0	30.0	5.0	13.0	4.0	24.0
55	8.0	14.0	5.0	27.0	11.0	14.0	6.0	31.0	5.0	13.0	4.0	25.0
60	8.0	14.0	5.0	28.0	12.0	14.0	6.0	31.0	5.0	14.0	4.0	25.6
65	9.0	15.0	6.0	29.0	12.0	15.0	7.0	33.0	6.0	15.0	5.0	26.0
70	9.0	15.0	7.0	30.0	12.0	16.0	8.0	33.5	6.0	15.2	5.0	27.0
75	10.0	16.0	8.0	31.0	13.0	17.0	8.0	35.0	7.0	16.0	6.0	28.0
80	11.0	17.0	8.0	32.0	14.0	17.0	9.0	36.0	7.0	17.0	6.8	29.0
85	12.0	17.0	9.0	34.0	15.0	18.0	10.0	38.0	8.0	18.0	8.0	31.0
90	14.0	18.0	11.0	37.0	17.5	18.0	11.0	41.0	8.0	18.4	9.4	31.4
95	16.0	19.0	13.0	39.0	20.0	19	13.0	44.8	9.0	19.0	12.0	34.2
99	21.0	20.0	16.0	45.0	23.8	20.0	17.8	48.8	15.2	20.0	16.0	40.2

*MDDI* Muscle Dysmorphic Disorder Inventory, *MDDI DFS* MDDI Drive for Size subscale, *MDDI AI* MDDI Appearance Intolerance subscale, *MDDI FI* MDDI Functional Impairment subscale, *M* Mean, *SD* standard deviation

Significant differences in the MDDI total score and the DFS and FI subscales were observed across gender minority groups (*p* < 0.001); no differences were observed for the AI subscale (Table 4). Specifically, transgender men scored significantly higher than gender-expansive participants and transgender women on the DFS subscale. Gender-expansive participants scored higher on the DFS subscale than transgender women. On the FI subscale, transgender women scored lower than transgender men and gender-expansive participants; however, no significant differences were observed between transgender men and gender-expansive participants. Finally, transgender men scored significantly higher on the MDDI total score than gender-expansive participants and transgender women, and gender-expansive participants scored higher than transgender women.

**Table 4:** Comparisons of Muscle Dysmorphic Disorder Inventory (MDDI) Total and subscale scores among gender-expansive people, transgender men, and transgender women in The PRIDE Study

	Groups			Kruskal-Wallis Test		post hoc Dunn’s test
	Gender Expansive People (a)	Transgender Men (b)	Transgender Women (c)	χ^2^	*p*	
	*Mdn* (IQR)	*Mdn* (IQR)	*Mdn* (IQR)			
MDDI DFS	7 (19)	10 (20)	5 (17)	197.15	< .001	b > a > c
MDDI AI	13 (16)	13 (16)	13 (16)	2.05	.358	–
MDDI FI	4 (16)	5 (16)	4 (12)	16.48	< .001	b, a > c
MDDI Total	27 (43)	30 (44)	24 (29)	87.50	< .001	b > a > c

*MDDI* Muscle Dysmorphic Disorder Inventory*, MDDI DFS* Drive for Size subscale, *MDDI AI* Appearance Intolerance subscale, *MDDI FI* Functional Impairment subscale

Overall, 6.5% of gender-expansive participants, 14.7% of transgender men, and 2.8% of transgender women had a total score in the clinically significant range (≥39), with these proportions reflecting significant group differences (*χ*^2^ (2) = 30.42, *p* < .001). Specifically, pairwise chi-square analyses indicated that a higher proportion of transgender men were in the clinical range than transgender women (*χ*^2^ (1) = 15.99, *p* < .001, OR = 5.92 [95% CI = 2.31, 19.44]). In addition, a higher proportion of transgender men, when compared with gender-expansive participants, were in the clinical range (*χ*^2^ (1) = 20.81, *p* < .001, OR = 2.50 [95% CI = 1.65, 3.78]). There was no significant difference between transgender women and gender-expansive participants in terms of clinically significant score ranges (*χ*^2^ (1) = 2.94, *p* = .086, OR = 0.42 [95% CI = 0.13, 1.06]).

## Discussion

In this study, we present, for the first time, norms of the MDDI in gender minorities from the United States. Consistent with our expectations, we found that transgender men reported the highest average MDDI total score, followed by gender-expansive people, then transgender women. The gender minority group differences in MDDI total score were driven largely by the DFS subscale and, to a lesser extent, the FI subscale. Notably, there were no significant differences in the AI subscale among the three groups. This study contributes to the scant literature on MD among gender minorities, providing norms for the MDDI and informing a broader understanding of the nature and degree of MD symptoms in gender minority populations in the United States.

### Transgender men

Transgender men reported the highest average MDDI total score and DFS and FI subscale scores. Additionally, transgender men were more likely to score above the clinical cutoff compared to transgender women and gender-expansive participants. These findings are in line with an Italian study by Amodeo and colleagues [11] that found that transgender men reported higher muscularity concerns than non-binary individuals, transgender women, and cisgender women. Transgender men were also found to report an equally high drive for muscularity compared to cisgender men, consistent with gender norms and body image ideals among men, including transgender men, that are commonly focused on muscularity [32]. Motivated in part by a desire to affirm one’s identity, transgender men may engage in traditionally masculine muscle-enhancing behaviors such as bodybuilding and fitness [33]. These activities may also involve specific efforts to modify one’s body shape to be more consistent with gender-specific body ideals, such that transgender men may work towards building a more muscular chest and reducing ‘feminine’ fat, especially around the hips [33]. Thus, relative to transgender women and gender-expansive people, the greater severity of MD symptoms among transgender men may be uniquely intertwined with the affirmation of the male identity and pressures to conform to masculine norms [34, 35]. Whereas traditional eating disorder symptoms oriented toward thinness and weight loss, along with the concomitant loss of menses and feminine body shape, are purportedly intertwined with the construction of a female identity [34], a hegemonic masculine identity is often conflated with the presence of muscularity [36]. As such, and in keeping with our findings, the affirmation of a male identity among transgender men may pose unique vulnerabilities with regard to muscularity-oriented psychopathology.

### Transgender women

We found that transgender women had the lowest average MDDI total score and DFS subscale score, which aligns with prior research [11]. This finding also accords with prior studies showing lower MD symptoms, particularly DFS, among cisgender women [11, 23]. Transgender women may experience lower muscularity concerns due to feminine body norms placing greater emphasis on thinness or on muscle tone versus bulk [12, 37]. As the MDDI was initially developed in men and the construct of MD more generally emphasizes muscle size versus definition or leanness, the items may have differential relevance for women. This may also explain the lower internal consistency of the MDDI total scale and the DFS subscale among transgender women relative to transgender men and gender-expansive people, respectively. Future work will need to be conducted to better understand potentially unique considerations regarding the nature and assessment of MD symptoms among transgender women. For instance, a future study could concurrently examine both drive for thinness and drive for muscularity among transgender women and transgender men to elucidate how the constructs may differentially relate to mental health concerns across these groups.

### Gender-expansive people

Overall, gender-expansive people reported average scores on the MDDI that were intermediate to those of transgender men and transgender women. We found a greater range and variability in MDDI scores in this group relative to transgender men and women, which is consistent with prior findings in this population, thus indicating heterogeneity in body image concerns, including in regards to overall body shape and specific body parts such as chest size and genitals [38]. Some gender-expansive people also expressed a drive to achieve an “androgynous or fluid” body ideal, or a balance of masculine and feminine traits [38]. This is consistent with the current finding that scores on the AI subscale did not significantly differ across the three gender minority groups. Moreover, gender-expansive individuals may be disproportionately exposed to certain social stressors, interpersonal violence, and psychological comorbidities (such as self-harm behaviors), relative to cisgender and binary transgender men and women, which in turn may elevate their risk for body image concerns [13]. As such, research on body image, disturbance to body image, and related risk and protective factors are warranted in this population.

### Comparisons to prior studies

Compared to the initial MDDI validation study in a selected sample of presumably cisgender men weightlifters [16], our community sample of TGE people reported qualitatively higher MDDI total scores (24.6–30.5 vs 18.8) and AI subscale scores (12.8–13.2 vs 6.1), but similar DFS (6.1–10.7 vs. 7.5) and FI (5.5–6.6 vs. 6.4) subscale scores. A prior German study of presumably cisgender populations recruited through fitness and bodybuilding groups found that 25% of men and 16% of women scored above the same MDDI clinical cutoff used in this study [23], which are qualitatively higher than the clinical cutoffs we found among a community sample of TGE populations. However, these comparisons with presumably cisgender populations should be interpreted with caution given that people participating in fitness, bodybuilding, or weightlifting may be at greater risk for MD symptoms than the general population.

### Gender minority stress framework

While gender minority populations may differentially experience MD symptoms, such as those oriented toward a DFS, we found no significant differences in AI. One explanation for this may be the shared experience of discrimination, stigma, and prejudice that TGE individuals encounter secondary to society’s intolerance of their gender identity, termed the gender minority stress framework [9, 39]. Specifically, gender minority individuals may face psychological distress due to societal identity invalidation, decreased social support, and increased discrimination [40–42]. Many TGE individuals experience mistreatment and violence, as evidenced by results from the 2015 U.S. Transgender Survey, in which 46% of the participants reported verbal harassment and 9% reported being physically attacked due to their gender identity in the prior year [43]. Experiences of gender dysphoria may also play a role in AI, particularly with regard to discomfort with one’s body, as reflected in the MDDI item “I hate my body.” Of note, some studies have shown a decrease in body image disturbance and eating disorder symptoms among individuals who receive gender-affirming health care [44, 45]; thus, examining the effect of gender-affirming health care on MD symptoms is an important area of future research.

### Limitations

Certain limitations of the current study should be noted. First, access to or engagement in gender-affirming health care was not known in the current sample. Second, participants in this study were predominantly White and highly educated; thus, results may not be generalizable to all gender minority people in the United States, particularly those from racial or ethnic minority backgrounds. Third, this study was cross-sectional and did not assess the onset or duration of MD symptoms. Future prospective, longitudinal studies are therefore recommended. Fourth, the current study only administered the MDDI, which is one of several MD symptom measures (e.g., the Muscle Dysmorphia Inventory [14], Muscle Appearance Satisfaction Scale [15]). Thus, we were not able to evaluate whether the MDDI is the best measure for assessing MD symptoms in gender minority populations. Fifth, we did not conduct a psychometric validation as part of the current study, and future research is needed to psychometrically evaluate the MDDI in gender minority groups. Sixth, although we used a previously published cutoff for clinical significance [23], this cutoff was not developed specifically in gender minority populations. Nonetheless, strengths of the study include a relatively large and unique sample of populations that have been understudied, particularly within the MD literature.

## Conclusions

We report, for the first time, MDDI norms among gender-expansive people, transgender men, and transgender women. Given the increasingly recognized health disparities that affect gender minority individuals, establishing normative data on MD symptoms will facilitate the ability for clinicians and researchers to interpret MDDI scores in these understudied populations. Future research will be needed to examine the MDDI in gender minorities clinically diagnosed with MD or MD measures tailored to specific gender minority populations. Nationally representative, population-based research with the MDDI is needed to better approximate the prevalence of MD and factors associated with MD in diverse communities. Future research could examine if TGE individuals differ on overall body image/size dissatisfaction versus body dysmorphic symptoms (e.g., genital size dissatisfaction) and potential cascading effects of these differences. Furthermore, the important intersections of sociodemographic factors – such as race, ethnicity, age, and socioeconomic status – as well as gender minority stressors on the nature and degree of MD symptoms and mental health outcomes among gender minority populations warrants additional study.

## Data Availability

Data from The PRIDE Study may be accessed through an Ancillary Study application (details at pridestudy.org/collaborate).

## Abbreviations

AI: Appearance Intolerance subscale
BMI: Body mass index
DFS: Drive for Size subscale
DSM-5: Fifth Edition of the Diagnostic and Statistical Manual of Mental Disorders
FI: Functional Impairment subscale
M: Mean
MD: Muscle Dysmorphia
MDDI: Muscle Dysmorphic Disorder Inventory
PRIDE Study: Population Research in Identities and Disparities for Equality (PRIDE) Study
SD: Standard deviation
TGE: Transgender and gender-expansive
